# Grants to Foster-Mothers

**Published:** 1921-01-01

**Authors:** 


### THE PUBLIC WELL-BEING.

### Grants to Foster-mothers.

### SCHEME TO DEAL WITH ILLEGITIMATE CHILDREN.

"Baby-farming," as it is sometimes called, or rather, the boarding out of children, chiefly of unmarried mothers, is still accompanied by dangers in this country which constitute a difficult problem both for the authorities and for health visitors, and for large numbers of social workers who carry on friendly ministrations in an individual capacity in the slums or working-class districts. Boarding-out places are difficult to control, and difficult to supervise, though it is only fair to say that scandals connected with "homes" of this kind are far less numerous than they were ten or twenty years ago. But anything that can be done to minimise the dangers connected with them and to safeguard the interests of the children is to be welcomed, and therefore we commend to public notice the very sensible systems put forward recently by the Medical Officer of Hackney in this matter.

Reporting on a proposed system of grants to foster-mothers, he points out that there is no doubt that great, and not seldom undeserved, hardship falls upon unmarried mothers who are compelled to work for a living, especially upon those whose occupation is domestic work as servants; or who are engaged in employments of other kinds which impose upon them the necessity of boarding out their babies entirely, or during the day when they themselves are at work. The wages earned are insufficient in many cases to enable them to make payments to foster-mothers and others undertaking the care of the babies, commensurate with the work done for, and the food required by, such infants. Inasmuch as this work is not undertaken on account of disinterested benevolence, but for profit, it follows that the payments should be in excess of the expenses incurred in providing milk and other foods so as to leave a balance as a recompense for the trouble and labour involved.

If this balance be insufficient to satisfy the foster-mother there will be ever present the temptation to enlarge the balance by diminishing the amount spent on milk and other foods, and, if the temptation be not resisted, the child may have to suffer through inadequacy of food, possibly through actual starvation. It is to meet such cases as these that supplementary payments to foster-mothers have been proposed. In this same connection some means might be found for the avoidance of so early a separation of mother and child as most commonly has to be endured. The Medical Officer suggests that there are several courses which might be adopted, either conjointly or separately.

A list might be made giving the names and addresses of all women known to be in the habit of receiving such children and classifying them so as to distinguish those who take in babies during the day and those who take them in entirely as boarders. The health visitors might assist in making out the lists and in subsequently visiting and reporting upon the foster-mothers, their homes and surroundings, their methods of feeding babies, sufficiency of food of a proper kind, general cleanliness, personal and domestic, and general cleanliness and appearance of babies and conditions as to health. Those only who proved satisfactory in all respects under the health visitor's investigations should be registered and those only should be recognised as worthy of receiving official aid. Such a register having been formed, mothers should be encouraged to send their babies to the registered foster-mothers rather than to others not on the register; and by judicious aid it might be ordained that charges at the recognised homes should not be more for mothers, but, if possible, less than the charges at those not on the register. This would encourage mothers to use the good homes in preference, and might result eventually in starving out the others.

Crèches to receive, during working hours, the babies of unmarried mothers while at work, and also to receive, as boarders, children of unmarried mothers who, on account of the nature of their work, are compelled to board out their children, should be under the complete control of the Child Welfare Committee, with a matron in charge, or a foster-mother, as a servant of the Committee. There should be several such institutions suitably placed so as to be accessible for all parts of the borough.

A number of day crèches in well-chosen spots for day workers whose mothers desired to board them out should be completely under the Committee's control; and the people serving in them should be appointed by the Committee.

This last scheme is the one the Medical Officer would prefer. If so desired the central establishment might be used also as a day nursery, but he is inclined to think there might be advantage in keeping the two classes of mothers apart.

It may be added that the Borough Council has not yet come to any conclusion regarding the grants of foster-mothers. But we hope it will deal with this important question in the same spirit of sympathetic and practical understanding as that displayed by its medical adviser.

